# Abortion rights are health care rights

**DOI:** 10.1172/jci.insight.171798

**Published:** 2023-06-08

**Authors:** Enze Xing, Rieham Owda, Charisse Loder, Kathleen Collins

**Affiliations:** 1 Medical Science Training Program,; 2 Department of Obstetrics and Gynecology, and; 3 Department of Internal Medicine, University of Michigan Medical School, Ann Arbor, Michigan, USA.

## Historical perspective on abortion in the United States

The practice of abortion is deeply rooted in American history. The practice of “restoring the menses” was prevalent among European colonists, Indigenous tribes, and enslaved Africans, with many women using herbal recipes shared by mothers, aunts, daughters, and sisters (1). Even health manuals that provided guidance were published. By the mid-18th century, premade abortifacients were available in New England and sold by traveling salesmen (1). Induction of miscarriage was considered part of a woman’s self-care regimen and was acceptable up to the point of “quickening,” when the fetus first kicks — only then was abortion considered illegal and immoral (2). The first laws banning the use of abortifacients after quickening were passed in the 1820s and 1830s, with the intent to protect women from potentially poisonous remedies for so-called “menstrual obstruction” and the men who coerced their use (1). By the 1840s, women received abortion care through mail-order pharmacy or by procedural specialists (3).

The American Medical Association (AMA) started the antiabortion movement in 1857 to wrest control of reproductive health from the purview of midwives. Criminalization of abortion at every stage, except for those deemed medically necessary by a physician, restricted clientele from the midwives and homeopaths who previously dominated maternal health and women’s health care (3). Female physicians were initially also accused of performing illegal abortion procedures to undermine their legitimacy as health care providers; however, by the 1890s, female physicians joined and even led the national anti-midwife, anti-abortion campaign (3). Finally, the AMA rallied support by claiming that decreasing birth rates from White Protestant families due to abortion access would result in overpopulation by minorities, especially “Indians,” Chinese, Mexicans, Blacks, and Catholics, a sentiment still echoed in “Great Replacement” conspiracy theories that continue to exist today (3). This movement led to a century of criminalized abortion in the United States, pushing women, especially women of color, to risk their lives by visiting unregulated underground providers, or by using bleach, turpentine, crochet hooks, clothing hangers, or chicken feathers at home (3).

One hundred years later, social activism refocused on women’s rights and spurred a pro-choice movement. In 1965, the Supreme Court’s ruling in *Griswold v*. *Connecticut* protected reproductive decisions in the form of contraception use under the implied right to privacy granted by the Constitution (4). Individual states also started reevaluating their stance on abortion, with Hawaii and New York legalizing the procedure in 1970, but there was no unified, federal decision regarding the legality of abortion bans. In 1969, Texas’s abortion ban was challenged as unconstitutional by attorneys Linda Coffee and Sarah Weddington, representing Norma McCorvey (Jane Roe in the ensuing court cases) against Henry Wade, McCorvey’s district attorney. Although a Texas district court ruled the state’s abortion ban was illegal, Wade affirmed he would continue to prosecute physicians who performed abortions. The case was eventually appealed to the US Supreme Court in the case *Roe v*. *Wade*. In a 7-2 decision, the Supreme Court struck down Texas’s abortion ban on January 22, 1973, citing a woman’s right to abortion was implicitly protected by the right to privacy in the 14th Amendment, once again legalizing the procedure for all Americans (5).

Despite the federal protection conferred by *Roe v*. *Wade*, for 49 years abortion access varied by state. Many states enacted mandatory 24- to 48-hour waiting periods and enforced ultrasonography before pregnant patients could undergo medical or procedural abortions (8). Additionally, anti-choice groups successfully lobbied for 1,381 increasingly severe abortion restrictions between 1973 and 2022, with some states adopting bans as early as 6 weeks after the last menstrual period (6, 7). On June 24, 2022, the Supreme Court ruled that the Constitution does not confer a right to abortion in *Dobbs v*. *Jackson Women’s Health Organization* (9). This landmark decision overturned the precedent set by *Roe v*. *Wade*, thus ending federal protection for abortion rights and allowing individual states to dictate abortion access for their residents. At the time of writing, 13 states have completely banned abortion, 5 states have gestational limits within the first or second trimester, and 7 states have bans temporarily blocked by district judges (10). Many of these bans allow no exceptions for rape, incest, or fetal anomaly incompatible with life, and some even refuse exceptions to save the life of the mother.

## Impact of abortion bans on pregnant patients

Abortion bans are commonly justified as laws to protect the unborn fetus, especially in cases of unintended pregnancy. However, these arguments neglect to consider the nuanced nature of reproductive care or consider the underlying causes that push women to seek pregnancy termination. Pregnancy is imperfect, and various complications that threaten the health and well-being of both the mother and fetus commonly arise. While criminalization of abortion will indeed affect women seeking to terminate an unintended pregnancy, it will also target a wide variety of patients seeking care for other reasons.

An immediate example are women with strongly desired pregnancies who require an abortion due to a life-threatening diagnosis. Pregnancy can be dangerous, and the United States currently possesses the highest rate of maternal mortality of high-income countries — the risk of dying from childbirth is 50–130 times greater than abortion (11–13). For patients experiencing severe medical conditions, abortion is not only safer than birth, it becomes a life-saving procedure. However, even for states that allow exceptions to preserve the life of the mother, the legal terminology is vague. How high and imminent must the risk of death be before treatment is allowed? Do patients who develop complications, such as pulmonary hypertension or cardiomyopathy, that incur a 20%–50% risk of death with ongoing pregnancy, qualify for treatment (13, 14)? What about women who are diagnosed with cancer during pregnancy? Due to the teratogenic nature of many oncological treatments, therapies may be withheld from these patients while pregnant. For these patients, the risk of death can be dramatically reduced by abortion and immediate cancer treatment; however, life-saving treatment may be delayed for months or even years. There is a myriad of similar situations, and until legal battles are fought concerning these details, uncertainty will lead providers to refuse and/or delay care for these complex cases.

Similarly, serious obstetric complications illustrate the complex decision-making process women must face regarding the termination or continuation of a pregnancy. One such example is previable preterm premature rupture of membranes (PPROM), defined as loss of amniotic fluid prior to 24 weeks of pregnancy. Previable PPROM complicates approximately 1% of pregnancies in the United States, resulting in a 50% risk of developing intrauterine infection, which can progress to sepsis and death without intervention (15). Previable PPROM also associates with neonatal morbidity and mortality, secondary to incomplete fetal development and complications of extremely premature birth, with fetal death occurring in approximately 32% of affected pregnancies. After thorough counseling with their health care providers, including weighing fetal and maternal risks, some women may choose to continue their pregnancy while others may choose to terminate. In a recent study, 57% of patients with a diagnosis of previable PPROM experienced significant maternal morbidity, even when expectantly managed (16). Abortion restrictions significantly affect the medical care that patients receive and prevent patients from accessing lifesaving, evidence-based care.

It’s important to consider that fetal anomalies are most commonly diagnosed in the mid-to-late second trimester. According to the Centers for Disease Control and Prevention, an estimated 3% of pregnancies are complicated by a fetal anomaly (10). Congenital malformations, such as CNS and chromosomal abnormalities, are the leading cause of infant mortality in the United States, accounting for 10% of intrauterine fetal demises and 20% of infant deaths (17, 18). Patients and their families are often considering multiple factors, including fetal risk, maternal risk, and the desire to reduce suffering, when making decisions following diagnosis of fetal anomaly. Given the increased risk of fetal death in utero in these scenarios, some women may decide to terminate their pregnancy instead of carrying a nonviable pregnancy and waiting for spontaneous intrauterine fetal demise to occur. A study of 53,000 pregnancies showed differential abortion rates, depending on the severity of the fetal anomaly. Mild anomalies, such as minimal renal pelvic dilation or pericardial effusions, did not associate with abortion, while 78% of women chose to terminate fetuses with severe anomalies, such as renal agenesis or anencephaly (19). Some women decide to terminate to alleviate any suffering that their fetus may endure. For others, the risk of remaining pregnant for the sake of a nonviable pregnancy is too high. This risk is important to take into account, because maternal mortality rates in the United States indicate childbirth is not innocuous, and abortion greatly reduces the risk of death from pregnancy (11–13). Patients should be empowered to choose the option that is best for them, their families, and the current pregnancy.

Unfortunately, even those experiencing miscarriage or spontaneous abortion will be negatively affected and even criminalized by abortion bans. Miscarriage, defined as the unexpected loss of a pregnancy before 20 weeks of gestation, is a common early complication that affects over 1 in 4 pregnancies (20). Pregnancy loss significantly traumatizes women physically and emotionally, and women undergoing miscarriage crucially need the care and support of their health care providers (21). While some instances of miscarriage can be expectantly managed, patients may also need medical or surgical management for complications such as incomplete uterine emptying, infection, or excessive bleeding (22). The best evidence-based medical management for spontaneous abortion is a combination of mifepristone and misoprostol, the same medications used in abortion care (13). However, abortion restrictions force providers to be hesitant about managing patients with these medications, as use of these medications may result in accusations of criminal activity despite providing best-practice care. Additionally, spontaneous pregnancy loss is clinically indistinguishable from medication-induced abortion, and patients presenting with bleeding in pregnancy or pregnancy loss are vulnerable to the threat of reporting, arrest, and detention, regardless of the cause of their symptoms (13). Despite the lack of legislation requiring reports of suspected self-managed abortion, health care providers have already been demonstrated to be more likely to report pregnant patients who are Black or low income (23). Between 2006 and 2020, there was a 3-fold increase in arrest, detention, and convictions secondary to pregnancy-related outcomes compared with 1973–2005. We have already seen instances of patients being reported by medical providers and being prosecuted in states, including Indiana and Texas (24, 25), that have enacted strict abortion restrictions. In the wake of *Roe v. Wade* being overturned and increased criminalization of abortion, we only expect the number of women criminalized based on their pregnancy-related health care choices to increase.

Patients will also have increased difficulty accessing appropriate surgical management of miscarriage when abortion bans are enforced. One of the best predictors for a physician providing the full spectrum of miscarriage management, including appropriate surgical intervention, is having had abortion care training as a resident (13). However, with the overturn of *Roe v*. *Wade*, 44% of current obstetrics and gynecology trainees in the United States are certain or likely to lack access to abortion training, with the number of trainees receiving abortion training predicted to drop from 92% to 56% (26). When abortions are criminalized, obstetrics and gynecology providers will no longer receive training in pregnancy termination, and this directly translates to lower quality of care for patients seeking termination or miscarriage management, especially in emergent situations. As health care providers become increasingly cautious in providing care for patients experiencing miscarriage due to fear of prosecution, patients have already experienced unbelievable horror stories. Numerous women have reported being denied medical and surgical interventions after presenting to their physician, and having to carry their dead fetuses for weeks, sometimes until they were actively febrile (27–30). One patient, who was also a health care provider, stated she “[fought] with the doctors for a while, but none of them would help me until I was actively sick. I was just dumbfounded. Especially as a nurse, no one comes into an E.R. and we wait to see how sick they can get” (28).

Overall, abortion bans will significantly affect both pregnancy-related and nonobstetric outcomes for pregnant women. If the United States bans abortion, maternal mortality associated with pregnancy-related causes is expected to increase 21%, with Black women incurring a 33% increase compared with 13% among White women (31). Shockingly, even more women are expected to die due to interpersonal violence. Women who are pregnant or recently postpartum are 16% more likely to be murdered than those who are nongravid (32). In fact, pregnant and postpartum women are more than twice as likely to die by homicide than bleeding or placental disorders and are often killed by an intimate partner (32). In short, abortion is an essential component of health care, and outlawing abortion will result in lasting effects on women’s health, including a significant increase in preventable death.

## Wider effects of abortion bans on the health care system

Legislation that restricts or bans abortion care can have far-reaching effects on the health care system and will result in disruptions of the lives of patients outside the realm of reproductive care. An illuminating example that has already emerged is pharmacists declining to fill medications associated with abortion. In the wake of the *Dobbs* decision, there have been multiple reports of patients being denied access to necessary medications, such as methotrexate, misoprostol, and mifepristone (33–37). While these medications are widely recognized as treatments for ectopic pregnancy, miscarriage, and induction of medical abortions, they are also routinely used in the management of chronic diseases, including countless autoimmune conditions, cancer, gastric ulcers, and Cushing’s disease. At the time of writing, the use of mifepristone has been banned in Wyoming, and a Texas judge may force the US FDA to withdraw its approval of mifepristone, affecting accessibility not only in states with abortion restrictions, but also in states where abortion is legal. The uncertainty surrounding these medications will be debilitating for patients with chronic conditions who rely on these prescriptions to keep their disease well controlled. Abortion restrictions will continue to negatively impact the health care system in a multitude of unpredictable ways, and the full consequences of these legislative decisions on patients will not be clear for many years.

## The role of health care providers in patient advocacy

What can medical professionals do in this critical time to support their patients? The first step is recognizing that many patients are afraid of becoming pregnant in this political landscape and will likely reach out to their trusted medical provider to discuss their concerns. It is important that we create a nonjudgmental space for patients to disclose these concerns and allow for a healthy discussion. Primary care providers will be on the front lines and provide reproductive health care, including contraception counseling, evaluation and diagnosis of pregnancy, and pregnancy options counseling. It will be essential to provide this care to all patients, but especially to those who do not want to become pregnant or those with multiple comorbidities that would make pregnancy life-threatening, as abortion access is no longer guaranteed. Providers will need to be knowledgeable about these topics and will also need to be aware of resources within the community to provide patients with the best care. We should trust that our patients know what is best for them and empower them to take charge of their reproductive health. Our duty in this time is to be advocates for our patients and support them in making the best decisions for themselves.

Next, we can ensure that care providers know about emergency care and are also aware of legal implications for patients. Emergency medicine providers are another group that will encounter patients navigating the spectrum of reproductive health and will see a variety of patients who may be experiencing early pregnancy, miscarriage, or even abortion complications. As of 2018, it was estimated that 14 out of every 100,000 emergency department visits for women 14 to 59 years old were related to induced abortion, a number that will likely increase as more abortion restrictions go into place. It is imperative that emergency medicine providers remain up to date on the management of early pregnancy and abortion complications to allow for prompt evaluation and intervention if necessary. It is important to remember that spontaneous and induced abortion are indistinguishable from each other but present with the same complications. Providers must keep this in mind to provide the necessary medical care to patients in a timely fashion and avoid reporting patients to authorities, leaving them vulnerable to prosecution. This burden will mostly be felt by people from historically marginalized communities and may exacerbate mistrust in the medical system (23).

It is important to note that emergency medicine providers are not the only health care providers that will interact with women accessing the health care system during a time of need. It is the duty of all medical providers to put aside personal values to prioritize the well-being of our patients.

## Consideration of harm reduction models in the United States

Restrictions on abortion do not lower abortion rates, indicating women will continue to seek mechanisms of pregnancy termination outside of the health care system. While it may no longer be possible for providers to directly aid patients in these circumstances, there are changes in practice that should be considered in order to provide some level of protection. Abortion care limitations are not unique to the United States, and international health care providers, including those in Indonesia, Uruguay, Argentina, Peru, Zambia, Nepal, Kenya, and Tanzania, have been able to implement harm reduction models that respect legal boundaries while maintaining a close eye on the health of their patients (38, 39). These models center on the self-administration of misoprostol as opposed to abdominal trauma or self-instrumentation, which leads to a reduction in maternal mortality (40, 41). During harm reduction consultations, women are given guidance regarding pregnancy options, including how to safely use misoprostol, and provided in-person and telephone follow-up opportunities to monitor for medical complications or ongoing pregnancy (39). These models have been shown to be effective in ensuring the safety of women undergoing pregnancy terminations. In fact, the robustly documented, nationwide Uruguayan model resulted in a stunning 29.4% drop in maternal mortality during the decade it was implemented (42). Of course, there are also legal and ethical implications of harm reduction to examine in the context of the United States. Harm reduction consultations may result in the arrest of the woman attempting self-induced abortion, as utilization of abortive medications becomes a legislative target, and especially if physicians are required to report disclosures of abortion attempts in the future (38). Furthermore, women of color and those of low-income backgrounds are more likely to experience unintended pregnancy and subsequent abortion, and are the most likely to incur the consequences of harm reduction models (38). However, the advantages provided by adopting a harm reduction model make it an important consideration moving forward in the current political climate.

## Conclusion

The United States has had a tumultuous relationship with abortion. Once practiced by women, for women, it became a weapon wielded against minorities and midwives, then legalized and utilized as an essential part of health care, and finally, abortion has been politicized and made inaccessible again. Abortion bans do not selectively affect women seeking abortions — they punish patients who suffer pregnancy complications, patients who experience severe medical comorbidities while pregnant, and even patients outside the scope of reproductive health who happen to require drugs associated with abortion. Nuances of many abortion cases make it such that any legislation dictating access to abortion care will inevitably have wide-ranging and unpredictable negative effects on both patients and the medical system. The exact ramifications of these legislative changes will not be clear for years. As health care providers once again navigate this restrictive and challenging landscape, it is important to consider one of the highest tenets of medical practice: patient autonomy. Medical professionals must examine their biases to continue providing patient-led, evidence-based care, regardless of personal opinion.

## Version 1 06/08/2023

Electronic publication
